# Ultrafast Dynamics of Rydberg Excitons and Their Optically Induced Charged Complexes in Encapsulated WSe_2_ Monolayers

**DOI:** 10.1021/acs.nanolett.4c06428

**Published:** 2025-04-30

**Authors:** Armando Genco, Chiara Trovatello, Vanik A. Shahnazaryan, Oleg Dogadov, Alisson R. Cadore, Barbara L. T. Rosa, James A. Kerfoot, Tanweer Ahmed, Osman Balci, Evgeny M. Alexeev, Habib Rostami, Kenji Watanabe, Takashi Taniguchi, Seth Ariel Tongay, Andrea C. Ferrari, Giulio Cerullo, Stefano Dal Conte

**Affiliations:** † Dipartimento di Fisica, Politecnico di Milano, Piazza Leonardo Da Vinci 32, Milano, 20133, MI, Italy; ‡ Department of Mechanical Engineering, Columbia University, New York, New York 10027, United States; ¶ Abrikosov Center for Theoretical Physics, Dolgoprudnyi, 141701, Russia; § Department of Physics, ITMO University, St. Petersburg, 197101, Russia; ∥ Cambridge Graphene Centre, University of Cambridge, Cambridge CB3 0FA, U.K.; @ Department of Physics, University of Bath, Bath BA2 7AY, U.K.; △ Research Center for Electronic and Optical Materials, National Institute for Materials Science, 1-1 Namiki, Tsukuba 305-0044, Japan; ∇ Research Center for Materials Nanoarchitectonics, National Institute for Materials Science, 1-1 Namiki, Tsukuba 305-0044, Japan; ▼ School for Engineering of Matter, Transport and Energy, Arizona State University, Tempe, Arizona 85287, United States

**Keywords:** Rydberg excitons, trions, semiconducting monolayers, ultrafast spectroscopy, transition metal dichalcogenides

## Abstract

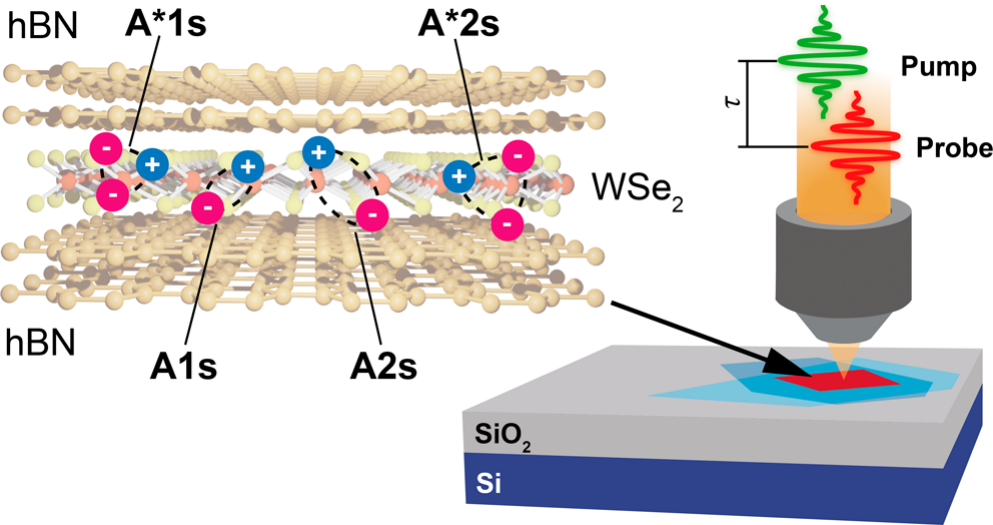

Quantum confinement and reduced dielectric screening lead to strong excitonic effects in atomically thin transition metal dichalcogenides (TMDs). Encapsulation of TMD monolayers in hexagonal boron nitride (hBN) unveils the excitonic Rydberg series below the free particle bandgap. The nonequilibrium response and the dynamics of these higher order exciton states and their multiparticle complexes remain almost unexplored. Here we use ultrafast pump–probe optical microscopy to measure the dynamics of excited-state (2s) excitons in hBN-encapsulated monolayer WSe_2_. 2s excitons form through an ultrafast relaxation process from high-energy states and exhibit longer decay dynamics than ground state excitons due to their higher spatial extension. We detect light-induced formation of 2s trions with significant oscillator strength and faster decay dynamics than 2s excitons, attributed to an intra-excitonic Auger effect causing an additional decay channel. Our results shed light on the dynamics of excited state excitons in TMDs and their interactions with free carriers.

Since their first observation,^1^ Rydberg states have triggered discoveries in atomic^2^ and condensed matter physics,^3,4^ such as revealing the quantized nature of atoms^5^ and allowing break-through experiments in quantum optics,^6^ owing to their large (hundreds of microns scale) Bohr radii.^7,8^ Monolayer (1L) TMDs^9,10^ host excitons with binding energies up to 500 meV,^11^ large oscillator strengths, and an associated series of excited excitonic Rydberg states below the free particle bandgap.^12^ Rydberg excitons in 1L-TMDs are detectable when the exciton linewidths are narrowed down to the homogeneous limit by hBN encapsulation^13^ and at cryogenic temperatures,^14,15^ deviating from the model system of a hydrogen atom for quantum numbers *n* < 3.^12^ The increased interaction strength of excited Rydberg excitons, propelled by their larger Bohr radius,^16^ can be further enhanced when they are strongly coupled to microcavity photons,^17,18^ potentially enabling quantum blockade.^19^

Strong Coulomb interactions in 1L-TMDs also stabilize many-body exciton complexes,^20^ such as trions, biexcitons,^21^ and other multiparticle states.^22−24^ These can interact with a Fermi sea of free charges generated by intrinsic^25^ or extrinsic^26^ doping, forming either three-particle bound states (trions) in the presence of low doping levels (when the free charge density is comparable to the exciton density),^20^ or exciton-polarons at elevated doping (>10^12^ cm^–2^).^27−29^ In the latter case, excitons are dressed by the electrons of the Fermi sea,^30−32^ resulting in Bose–Fermi mixtures, crucial for studying correlated many-body systems.^33^ Excited state trions, i.e., charged complexes of excitons at high Rydberg states, have been observed in the static optical response of 1L-TMDs.^34−40^ In gate-controlled optical experiments performed on W-based 1L-TMDs^36,40^ the binding energies of 2s positive and negative trions have been determined in n-doped and p-doped systems. Like the more studied ground state trions, also 2s trions gain oscillator strength at the expense of the 2s neutral excitons at increasing doping densities.^40^ While 1s trions exhibit binding energies an order of magnitude lower than the typical energies of 1s excitons,^40^ 2s trions have binding energies ranging between 15 and 30 meV,^36,37,40^ comparable to those of the corresponding 2s neutral excitons and of 1s trions. Moreover, the linewidth of 2s trions exhibits additional broadening with respect to the 1s trions.^40^

The nonequilibrium optical response of 1L-TMDs has been an object of intense study since their discovery.^41−46^ Despite the large amount of work on exciton dynamics, almost all ultrafast spectroscopy experiments focused on the investigation of neutral^42^ and charged excitons^47^ in their ground state. The dynamics of excited neutral Rydberg states is almost unexplored,^48^ while those of their charged complexes have never been investigated.

Here, we perform femtosecond transient reflection microscopy (Figure 1a) on hBN-encapsulated 1L-WSe_2_ at cryogenic temperatures (*T* = 8 K), combining both broadband detection (i.e., 1.6–1.9 eV) and high temporal resolution (∼50 fs), and measure an immediate formation of 2s excitons (within our temporal resolution) after photoexcitation above the free-particle bandgap (∼2 eV).^49^ By comparing the decay dynamics of 1s and 2s neutral excitons, we find a longer relaxation times for the latter, in agreement with calculations.^50^ We attribute the longer relaxation time to the different spatial extents of the 1s and 2s exciton wave functions. Our measurements also show an ultrafast all-optical generation of excited state (2s) trions. The photoinduced absorption signal of 2s trions is red-shifted by ∼20 meV with respect to the 2s exciton peak, and decays about twice faster than 2s excitons, as a result of an intra-excitonic Auger scattering process. Our findings clarify the nonequilibrium physics of excited state excitons and their many-body complexes in 1L-TMDs.

**Figure 1:**
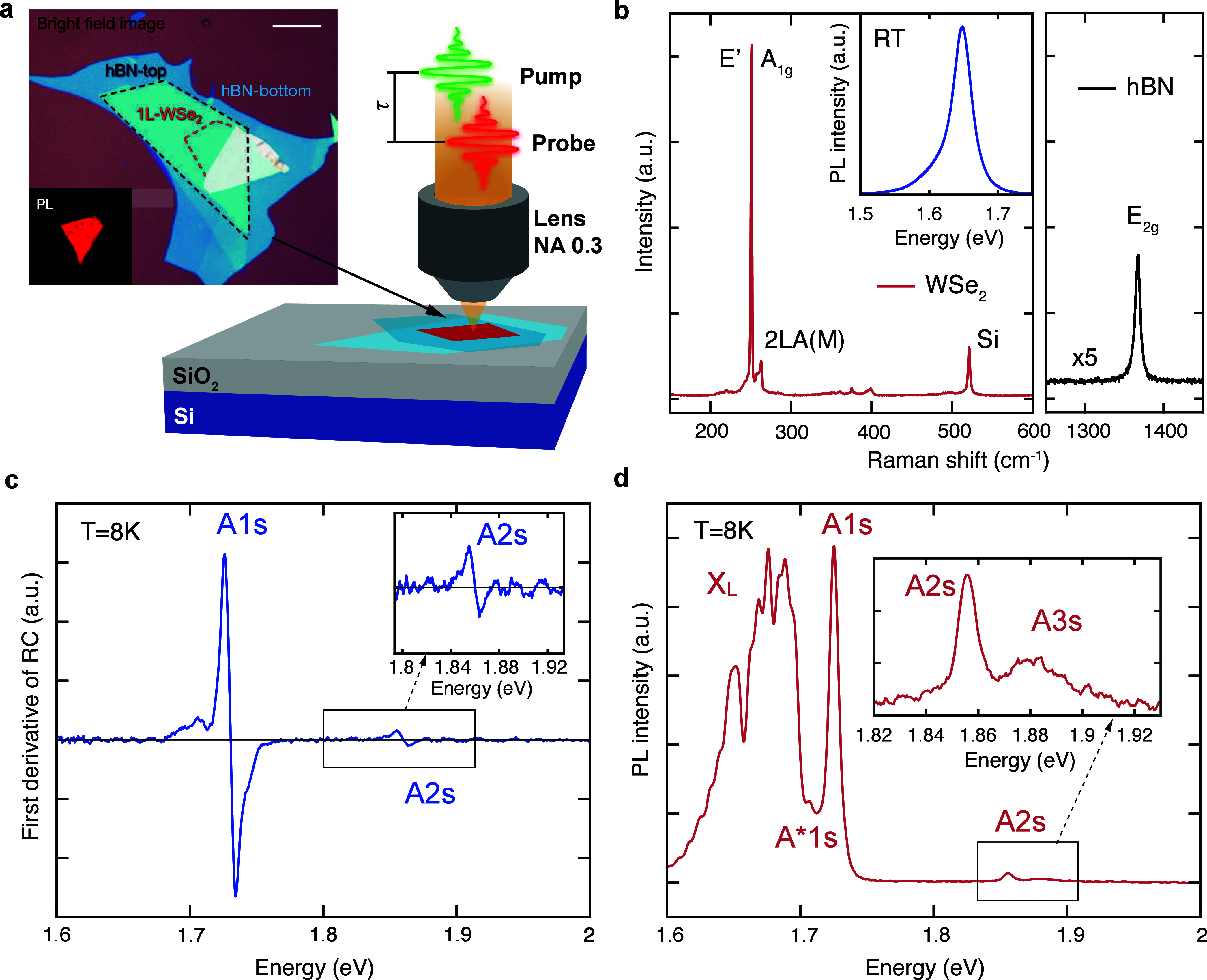
**a,** Schematic view of the sample under a microscopy setup for ultrafast pump–probe spectroscopy. Inset: bright field and PL microscope images of 1L-WSe_2_, outlined by the red dashed line, encapsulated in hBN. Scale bar: 10 μm. **b,** Raman spectrum of 1L-WSe_2_ (red line) and hBN (black line) at RT. Inset: PL spectrum at RT showing the A1s exciton peak of 1L-WSe_2_ at ∼1.65 eV. **c,** First derivative of the static RC spectrum at 8 K, showing A1s and A2s (zoomed in the inset) excitonic states. **d,** Low-temperature (8 K) PL spectrum of hBN/1L-WSe_2_/hBN excited with a CW 532 nm diode laser, displaying a bright A1s exciton peak and a weaker signal from excited Rydberg states (A2s, A3s) at higher energies (zoomed in the inset). X_L_ denotes the emission peaks from the localized and dark states of 1L-WSe_2_.

Our sample consists of a mechanically exfoliated 1L-WSe_2_ encapsulated between two hBN layers on SiO_2_/Si.^9^ More details on the fabrication can be found in Supporting Information. An optical image of the sample is shown in Figure 1a. Encapsulation in hBN narrows the exciton line width to approach the homogeneous limit (∼4 meV at 4 K^14^), as it ensures the isolation of the monolayer from external impurities and improves the conformation with respect to the use of rougher substrates, such as SiO_2_,^13^ eliminating blisters of trapped interfacial contamination.^51^

Figure 1b plots the Raman spectrum of hBN/1L-WSe_2_/hBN at room temperature (RT). For 1L-WSe_2_, the E′ and A_1g_ modes are merged in a single band ∼251 ± 0.2 cm^–1^, while 2LA(M) is ∼258 ± 0.2 cm^–1^ and A(M) is ∼263 ± 0.2 cm^–1^, as expected for 1L-WSe_2_.^21^ The hBN flake has a Raman mode ∼1367 ± 0.2 cm^–1^ as expected for multilayer hBN (ML-hBN).^52^ The uniform photoluminescence (PL) image in the inset of Figure 1a demonstrates the homogeneity of encapsulated 1L-WSe_2_. The inset of Figure 1b plots the RT PL spectrum of the sample, with a peak ∼1.65 eV corresponding to the neutral A1s exciton.^21^

Figure 1c shows the first derivative of the reflectance contrast (RC) spectrum taken at 8 K, with RC = (*R*_sub_ – *R*_1L_)/*R*_sub_, where R_1L_ and R_sub_ are the reflectivity of the sample with and without 1L-WSe_2_, respectively. We attribute the derivative features centered ∼1.725 and 1.86 eV to the absorption peaks of the 1s and 2s excitonic Rydberg states (the inflection point indicates the peak energies). The shoulder at ∼1.695 eV, lower in energy compared to the 1s exciton, is related to the 1s trion, A*1s.^53^ Its oscillator strength is ∼10 times lower compared to that of A1s,^40^ confirming that the sample is close to the neutral doping level. The PL spectrum (Figure 1d) further proves the low doping (<5 × 10^11^ cm^–2^),^40^ as the neutral exciton peak is more intense compared to highly doped samples where the trion emission completely dominates the PL spectrum.^54^ At lower energies several other peaks appear (X_L_), attributed to dark states,^55^ biexcitons,^54,56^ localized excitons,^57^ and defect states.^58^ A weaker PL signal from excited Rydberg states (A2s and A3s) is also observed at higher energies (zoomed in the inset of Figure 1d).

We use broadband optical pump–probe microscopy^46^ to study the transient optical response of hBN-encapsulated 1L-WSe_2_. A conceptual scheme of the experiment is shown in Figure 1a. Figures 2a,b plot the differential reflectivity (Δ*R*/*R*) maps as a function of probe photon energy and pump–probe delay in the spectral range of A1s and A2s excitonic transitions, respectively, for 2.34 eV excitation (above the ∼2 eV free-particle gap^49^).

**Figure 2:**
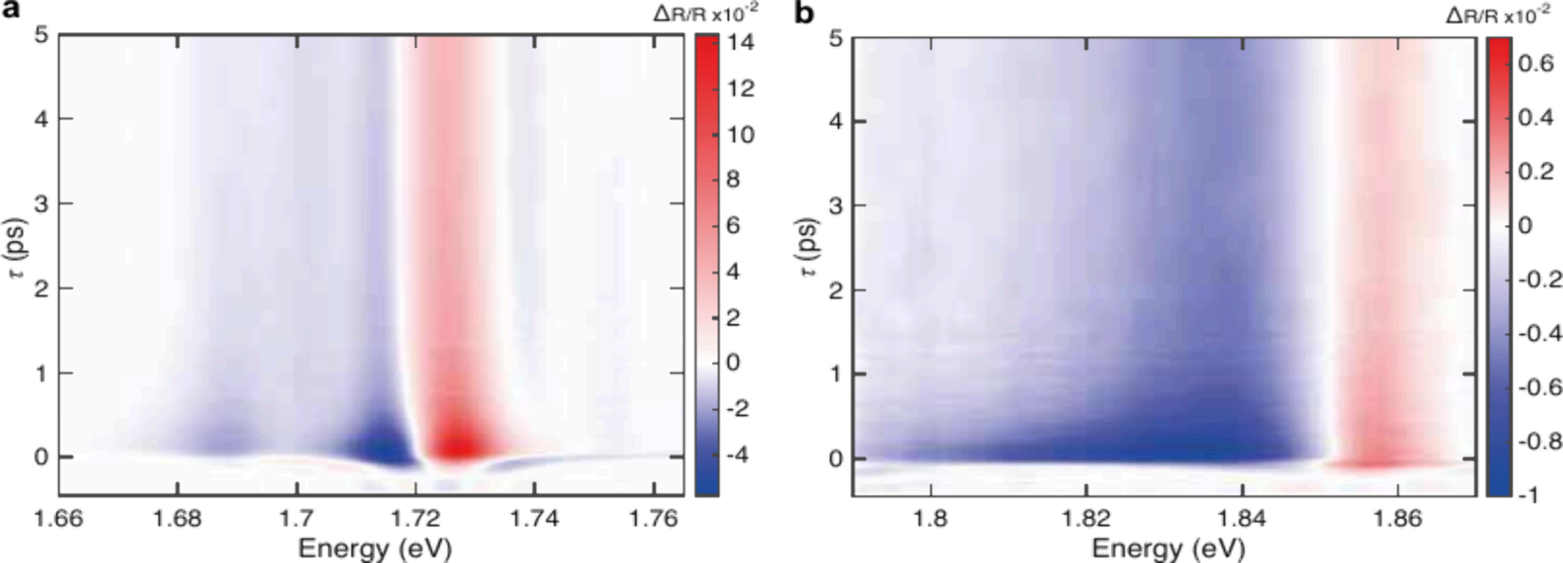
**a,b,** Color maps of Δ*R*/*R* in the spectral window of (a) A1s and (b) A2s excitons in hBN-encapsulated 1L-WSe_2_ as a function of pump–probe delay τ and probe photon energy. The incident pump fluence is ∼4 μJ/cm^2^.

Immediately after time zero, Δ*R*/*R* shows mainly positive signals around the A1s and A2s energies, with negative features on the low-energy side of each peak. References (59 and 60) reported that, for excitation above the bandgap, the energy renormalization of the A1s excitonic resonance is negligible, whereas Pauli blocking and linewidth broadening effects are the main processes responsible for the shape of the Δ*R*/*R* spectrum. For A1s, a dip appears at lower energy compared to the neutral exciton, at ∼1.69 eV (Figure 3a). The ∼35 meV energy difference between this feature and A1s matches the splitting between the trion and neutral exciton in doped 1L-WSe_2_.^40,61^

The A2s Δ*R*/*R* spectra (Figure 3b) show more strongly asymmetric shapes, with a small positive signal and a broad and pronounced negative signal extending on the lower energy side of the excitonic resonance. To understand how transient modifications of the excitonic peaks affect the Δ*R*/*R* signal, we retrieve the nonequilibrium absorption spectrum (α) by exploiting a Kramers–Kronig constrained variational method.^46,59^ The optical interference effects caused by the multilayer structure of the sample are taken into account by using the transfer matrix method (TMM) (Supporting Information). The results of this analysis are in Figures 3c,d, where the static and the nonequilibrium α spectra are reported. After 0.2 ps, the A1s peak is quenched and slightly blue-shifted (∼1 meV), as an effect of Pauli blocking and Coulomb interactions.^62^ The negative feature ∼1.695 eV in the Δ*R*/*R* map can be related to the occurrence of a photoinduced A*1s trion absorption, red-shifted by ∼35 meV from the neutral exciton.

**Figure 3:**
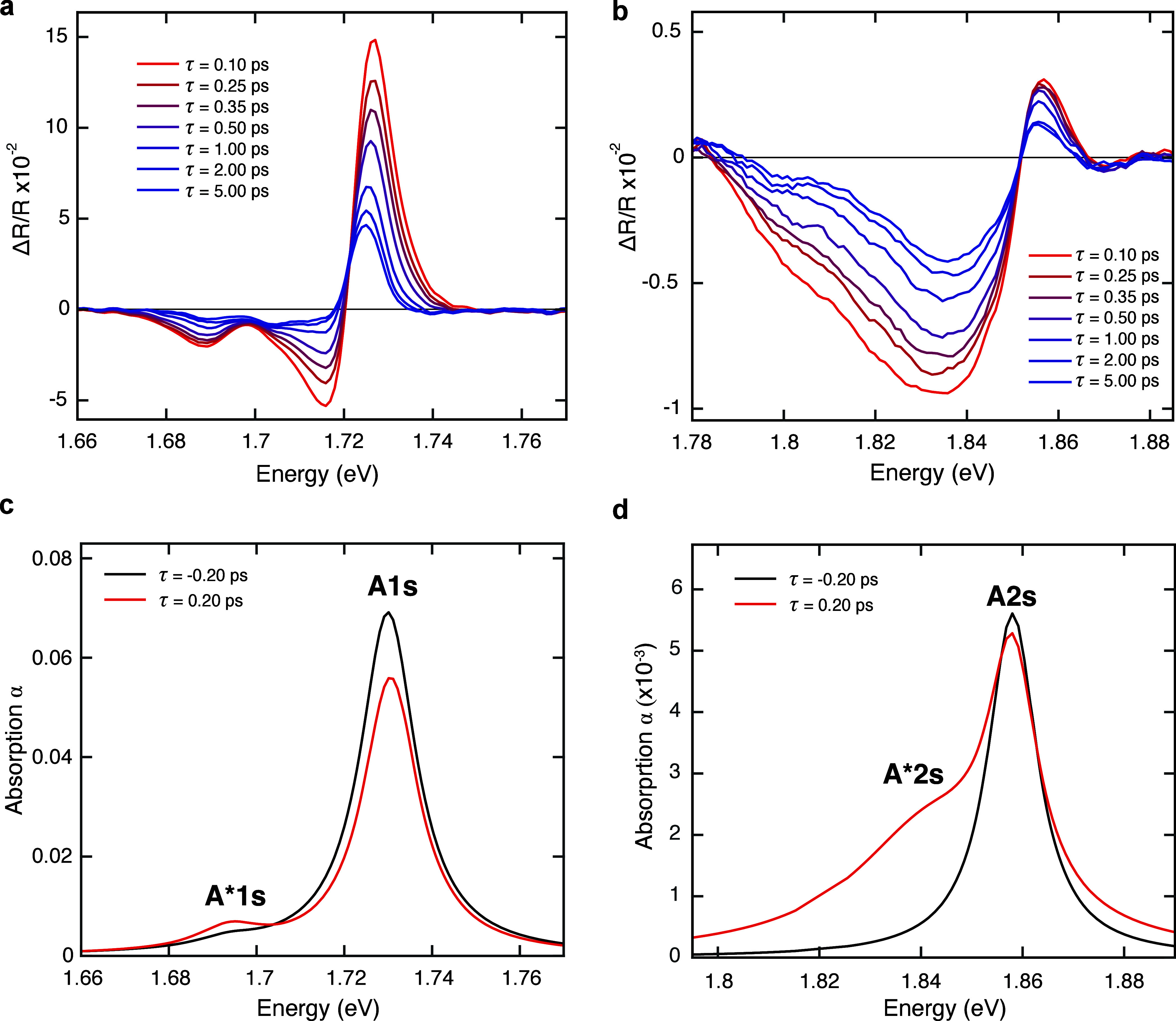
(a, b) Δ*R*/*R* spectra measured around the spectral region of A1s and A2s at different delay times. (c, d) Corresponding absorption spectra retrieved from the Δ*R*/*R* energy traces before photoexcitation (black lines) and at 0.2 ps delay (red lines).

Figure 3d shows that the A2s resonance is less quenched than A1s, while an ∼30 meV broad and prominent absorption feature, red-shifted by ∼20 meV, appears after excitation. We relate this to the formation of optically induced 2s trions (A*2s). The optically induced excited trion oscillator strength reaches almost half the intensity of the neutral A2s peak, being much higher than its ground state counterpart, probably because of the larger spatial extent and its stronger interactions with the Fermi sea of free carriers.

By comparing the normalized temporal dynamics of A1s and A2s (Figure 4a), tracking the change of the integrated intensity extracted from the calculated dynamic absorption spectra, we observe the simultaneous formation (within our ∼50 fs temporal resolution) of the transient optical response measured at the energies of 1s and 2s excitons. The extremely fast exciton formation dynamics can be assigned to a phonon-mediated exciton cascade process^44,63,64^ from higher to lower energy excitonic states. Upon photoexcitation above the quasi-particle gap, electron–hole (e-h) pairs lose their energy by emitting phonons and relax down to the ground state (Figure 4b). Differences in 1s and 2s exciton formation dynamics cannot be appreciated because of the limited temporal resolution of our experiment.

**Figure 4:**
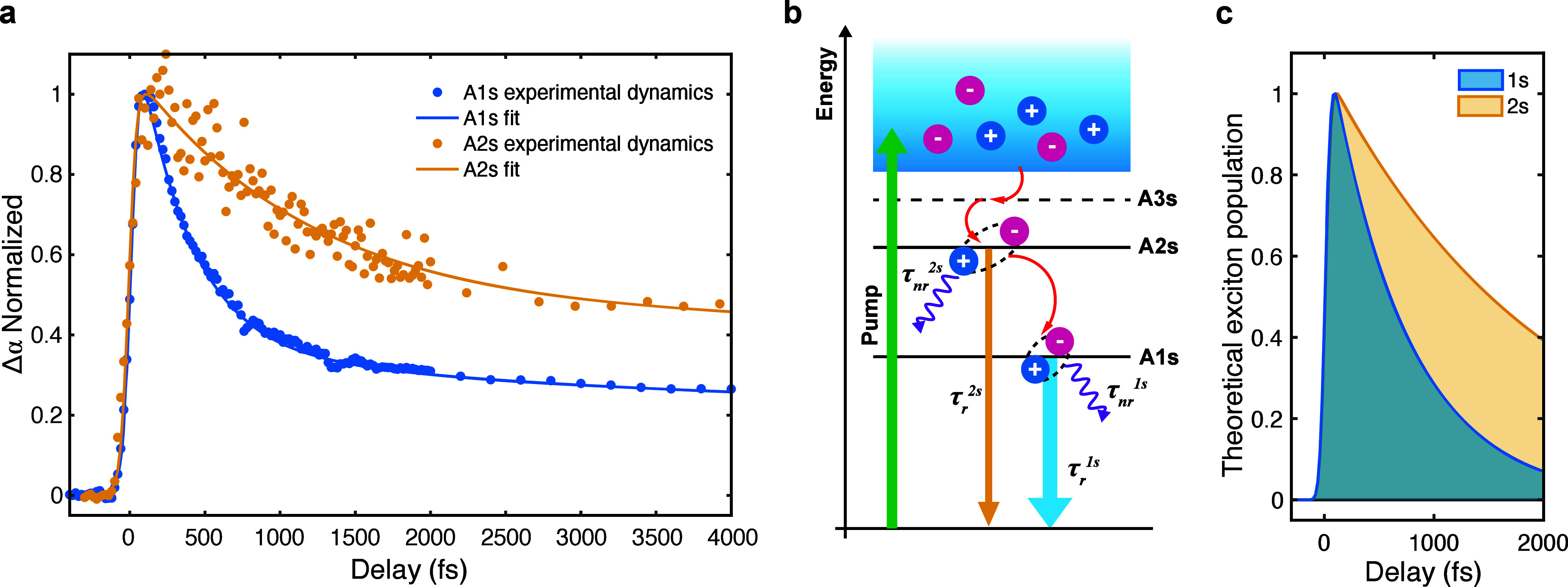
(a) Normalized transient variation of exciton-integrated oscillator strength extracted from the line-shape analysis of the 8 K pump–probe experiments, for Rydberg states A1s (blue dots) and A2s (dark yellow dots) in 1L-WSe_2_. The solid lines are multiexponential fits of the data. (b) Relaxation pathways for A1s and A2s excitons, depicting the exciton cascade formation process. The straight arrows pointing downward represent the radiative decay rate of the excitonic species, while the wavy arrows represent the nonradiative relaxations. Larger arrow widths stand for faster decays. (c) Shaded plots of the simulated exciton population dynamics extracted from the theoretical exciton decay rate for A1s (blue) and A2s (dark yellow).

The two species show different decay dynamics: both curves display a double-exponential decay, within the temporal window explored in this experiment (Figure 4a), much slower for A2s compared to A1s. The ultrafast optical response of 1L-TMDs on the sub-picosecond time scale is dominated by several scattering mechanisms. Optically excited excitons at high energy can radiatively decay to the ground state once their center-of-mass momentum is close to zero.^65,66^ Alternatively, they can relax toward dark excitonic states within the same valley by emitting optical or acoustic phonons^67,68^ or scatter to another valley upon absorbing large-momentum phonons forming momentum-indirect excitons.^69^ On a tens of picosecond time scale, the transient optical response is determined by other slower processes such as the scattering between excitons and defects^70,71^ and heating effects.^72^

We fit the curves using a multiexponential function convoluted with a Gaussian representing the instrumental response function^73^ (see details in the Supporting Information). We find that, for A1s, the fast decay time is τ_*A*1s_^fast^ = 0.37 ± 0.02 ps, while for A2s it is τ_A2s_^fast^ = 1.02 ± 0.11 ps. The second slow decay component is also longer for A2s excitons compared to A1s (∼95 ps versus ∼20 ps), but we focus our analysis only on the fast decay that is directly related to the relaxation processes of bright excitons, not involving slow interactions with the lattice. The experimental τ_A2s_^fast^/τ_A1s_^fast^ is ∼2.75, slightly lower compared to previous results of four-wave mixing experiments on hBN-encapsulated 1L-WSe_2_.^48^ To explain the different dynamics of A1s and A2s, we calculate the exciton decay rates including both radiative and nonradiative contributions. The overall fast decay rate is 1/τ_*i*_ = 1/τ_*i*_^*r*^+1/τ_*i*_^nr^, where *i* = {A1s, A2s}, and τ_*i*_^r[nr]^ is the exciton radiative [nonradiative] decay time. We attribute the nonradiative decay primarily to scattering between excitons and acoustic phonons. Our calculations neglect the scattering with optical phonons, considering the low temperature.^74^ At 8 K we get τ_*i*_^nr^ = 2.52 ps for both 1s and 2s exciton states. Using the dipole matrix element of interband transitions to fit simultaneously the dynamics of both 1s and 2s excitons, we obtain τ_A1s_^r^ = 0.9 ps and τ_A2s_^r^ = 9.7 ps, respectively (see Supporting Information for details). The ratio between the two radiative times τ_A2s_^r^/τ_A1s_^r^ ∼ 10 is determined by the probability density of exciton wave functions at the origin, i.e., when e and h positions overlap.^75^

Figure 4c shows the theoretical dynamics of the excitonic populations using the decay rates calculated above. These qualitatively reproduce the experimental dynamics within the first ps. The calculated ratio of the two fast lifetimes, including both radiative and nonradiative contributions, is τ_A2s_/τ_A1s_ ∼ 3, close to the experimental one for the fast decay times.

The longer decay dynamics of A2s compared to A1s is mainly explained by taking into account the larger Bohr radius of the excited Rydberg states,^12^ as suggested by ref (76). For higher quantum numbers *n*, the Rydberg exciton radiative decay time increases due to decreased recombination probability, linked to the more extended exciton orbital wave function.

The formation time remains instantaneous for A2s if the excitation energy is tuned in resonance with the 1s states (see Figure S2 in Supporting Information), confirming that A1s and A2s share the same excitonic ground state. This could also explain the intense upconverted PL from A2s, when pumping in resonance with A1s, previously observed in this system.^77^

Figure 5a reports the dynamics of A1s and A*1s. On a ps temporal window, the trion signal decays on a slightly longer time scale (τ_A*1s_^fast^ ∼ 0.5 ps), with respect to the neutral exciton, confirming the trend previously observed in time-resolved PL measurements on 1L-TMDs,^78^ but also on group III–V^79^ and II–VI^80^ semiconductor quantum wells. Reference (81) reported that the nonradiative decay of trions is slower with respect to that of A1s. The slow relaxation and diffusion dynamics of A*1s can be attributed to the strong interaction with phonons and the larger trion mass.^81^

The formation dynamics of the A*1s trion (i.e., the buildup dynamics of the trace in Figure 5a) displays the same time scale of the neutral exciton.

**Figure 5:**
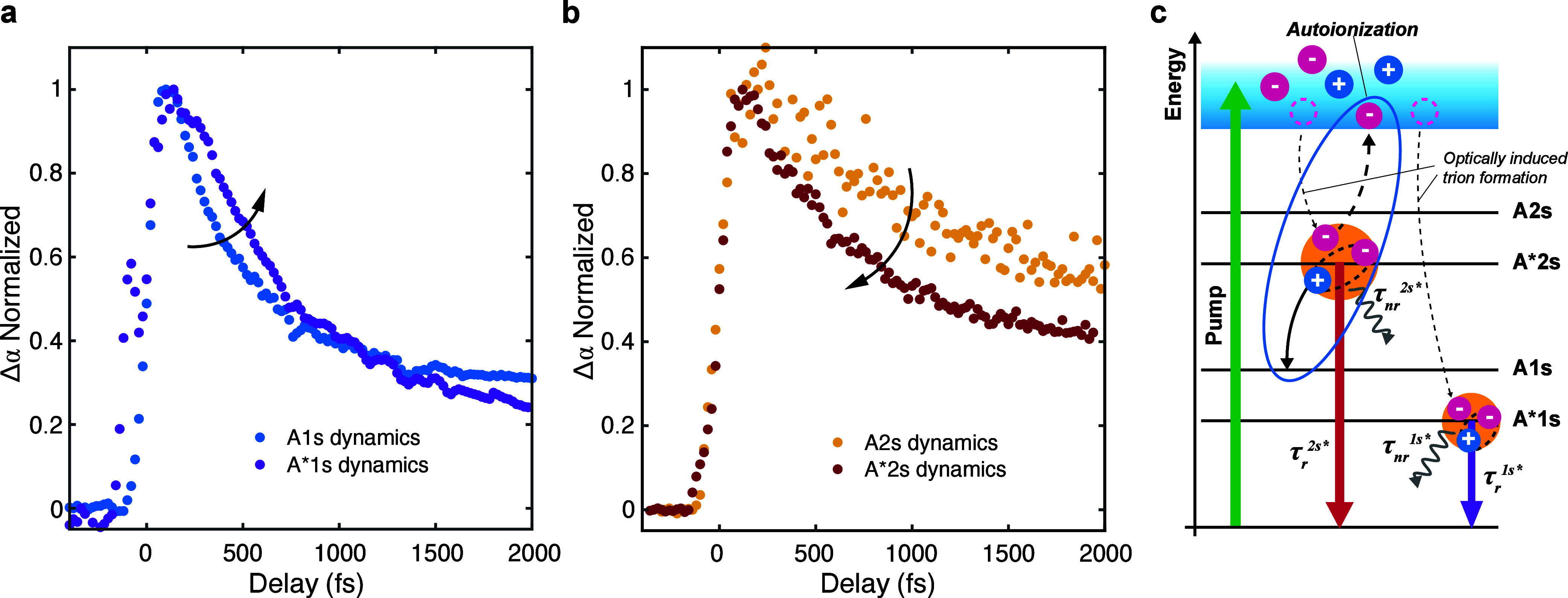
**a,** Normalized transient variation of integrated oscillator strength for A1s excitons (blue dots) and A*1s trions (purple dots). **b,** Normalized transient variation of integrated oscillator strength for A2s excitons (dark yellow dots) and A*2s trions (red dots) . **c,** Sketch of formation and relaxation processes for A*1s and A*2s charged excitons. Trions form owing to the optically induced free charges which are captured by excitons. The solid arrows represent the radiative and nonradiative decay rate of the excitonic species. The blue ellipse shows the additional decay pathway for A*2s due to autoionization.

Previous two-color pump–probe experiments performed on a different TMD (i.e., 1L-MoSe_2_ without hBN encapsulation) reported a slower time scale for trion formation, ranging from hundreds of fs^82^ to a few ps.^47^ This sample-dependent variation of time scales seems to indicate that extrinsic properties of the semiconductor, such as density of defects, disorder, and interactions with the substrate, might affect the formation dynamics of these excitonic species. Reference (47) showed that, by tuning the excitation energy across the inhomogeneously broadened exciton resonance, the rise time of the trion dynamics significantly increases. The change of the dynamics was explained in terms of the occurrence of a mobility edge, which separates localized and delocalized excitons in a disorder potential. Localized excitons are trapped by disorder- and impurity-induced potential. Their interaction with free carriers is reduced with respect to the delocalized excitons, which are characterized by high mobility and high probability to trap free charges. The sub-100 fs trion formation time observed in our experiments seems to confirm that trions in low disorder hBN-encapsulated 1L-TMDs are formed as a consequence of the trapping of free carriers by delocalized excitons.

For trion formation in semiconductors, 2- and 3-particle processes were proposed as possible mechanisms.^83^ The former process can be described as coalescence between bound excitons and free carriers, whereas the latter is a 3-body process in which the trions are formed directly from an unbound e–h plasma. Since the strong e–h Coulomb interaction in 1L-TMDs leads to an almost instantaneous formation of bound excitons upon photoexcitation of free carriers, and the A*1s signal does not show a significantly delayed formation with respect to the pulse-width-limited A1s rise time, the trion formation mechanism in 1L-TMDs is predominantly a 2-body process. Since our sample is very close to the neutrality condition, the free carriers trapped by the excitons are directly photoinjected by the pump pulse. Only a fraction of photoexcited carriers will relax to the ground state, while the remaining ones will be trapped by excitons forming three-particles states. A similar formation time scale (i.e., <100 fs) for trions was observed in carbon nanotubes^84^ and ascribed to a trapping of free carriers by bound excitons, with a negligible contribution of three-particle processes.

Figure 5b plots the dynamics of A2s and A*2s. The A*2s trion forms instantaneously (i.e., within 50 fs), confirming that the trapping of free carriers by 2s excitons is a very fast and efficient process. Focusing on the ps temporal window, the relaxation dynamics of 2s excitons and trions follows an opposite trend with respect to that observed for their ground state counterparts: the A*2s trion relaxes faster than the A2s exciton. The difference of decay dynamics for Rydberg excited trions versus neutral excitons, compared to their A1s/A*1s counterparts, can be attributed to the presence of an additional nonradiative relaxation channel due to the autoionization effect^85^ (or *intra-excitonic* Auger process) affecting only charged Rydberg states, and causing a further broadening of their linewidth.^40^ In this process, an excited state bound to an additional electron relaxes to the excitonic ground state, releasing excess energy that is transferred to another electron, causing it to become unbound. (Figure 5c). Therefore, the decay of A*2s, much shorter than that of A2s (0.65 ps vs 1.02 ps), is limited by the fast relaxation channel due to autoionization. We stress that autoionization can only occur for excited trion states: this Auger-like process cannot happen for 1s trions, because no lower excitonic bound state is available.^40^

In conclusion, upon photoexcitation of hBN-encapsulated 1L-WSe_2_, we observed the ultrafast relaxation of A1s and A2s Rydberg excitons and the all-optical formation of A*1s and A*2s trions. We modeled the ultrafast relaxation times of neutral excitonic species, and we attribute the slower relaxation of A2s compared to A1s to their larger Bohr radius. We observed pulse-width-limited formation dynamics of both trion species, as a consequence of a fast and efficient trapping process of free carriers by neutral excitons. In addition, we detected a faster decay of excited trions compared to neutral A2s excitons, explaining this as a result of an intra-exciton Auger process, leading to an additional relaxation pathway for A*2s. Our time domain experiments confirm previous arguments based only on the static analysis of exciton line widths.^40^ The rich behavior of Rydberg excitons unveiled in our work offers novel insights on the ultrafast dynamics and the many-body physics of excitons in 1L-TMDs. Our work showcases the possibility of directly tracking and manipulating excited Rydberg exciton species on ultrafast time scales, opening avenues for further fundamental studies and applications.

## Data Availability

The data that support this study will be made available in a public repository upon publication.

## Supporting Information Available

Sample preparation, optical measurements, transfer matrix analysis, theoretical calculations, multiexponential fits of the exciton and trion dynamics; additional pump–probe experiments (PDF)
