# Supplementary material for: Ultrafast Dynamics of Rydberg Excitons and Their Optically Induced Charged Complexes in Encapsulated WSe2 Monolayers
Source: Nano Lett. 2025 Apr 30;25(19):7673–81. doi: 10.1021/acs.nanolett.4c06428 (PMC12082702; doi:10.1021/acs.nanolett.4c06428)
Supplement: Supplementary file 1 — nl4c06428_si_001.pdf [file nl4c06428_si_001.pdf]

Ultrafast dynamics of Rydberg excitons and their optically-induced charged complexes in encapsulated WSe_2 monolayers: Supporting Information

Armando Genco,[*,†] Chiara Trovatello,[†,‡] Vanik A. Shahnazaryan,[¶,§] Oleg Dogadov,[†] Alisson R. Cadore,[∥] Barbara L. T. Rosa,[∥] James A. Kerfoot,[∥] Tanweer Ahmed,[∥] Osman Balci,[∥] Evgeny M. Alexeev,[∥] Habib Rostami,[⊥] Kenji Watanabe,[#] Takashi Taniguchi,[@] Seth Ariel Tongay,[△] Andrea C. Ferrari,[∥] Giulio Cerullo,[†] and Stefano Dal Conte[*,†]

†*Dipartimento di Fisica, Politecnico di Milano, Piazza Leonardo Da Vinci 32, Milano, 20133, MI, Italy*

‡*Department of Mechanical Engineering, Columbia University, New York, 10027, NY, USA*

¶*Abrikosov Center for Theoretical Physics, Dolgoprudnyi, 141701, Russia*

§*Department of Physics, ITMO University, St. Petersburg, 197101, Russia*

∥*Cambridge Graphene Centre, University of Cambridge, Cambridge, CB3 0FA, UK*

⊥*Department of Physics, University of Bath, Claverton Down, Bath, BA2 7AY, UK*

#*Research Center for Electronic and Optical Materials, National Institute for Materials Science, 1-1 Namiki, Tsukuba 305-0044, Japan*

@*Research Center for Materials Nanoarchitectonics, National Institute for Materials Science, 1-1 Namiki, Tsukuba 305-0044, Japan*

△*School for Engineering of Matter, Transport and Energy, Arizona State University, Tempe, 85287, AZ, USA*

E-mail: armando.genco@polimi.it; stefano.dalconte@polimi.it

Sample preparation

Multilayer (ML) hBN flakes on SiO_2/Si substrate are prepared by micromechanical cleavage of bulk hBN, grown at high pressure (4.5 GPa) and temperature (1500 °C) in a barium-BN solvent.[1] 1L-WSe_2 on PMMA (Spin coated PMMA A8 on glass) is prepared by micromechanical cleavage using Nitto-tape[2] of flux-zone grown[3] bulk WSe_2 onto PMMA at 80 °C. Upon identification of all suitable layered materials by optical microscopy,[4] ML-hBN/1L-WSe_2/ML-hBN is then assembled using a dry-transfer method, as described in Ref. 5. First, a polycarbonate (PC) membrane on a polydimethylsiloxane (PDMS) stamp (as mechanical support) is brought into contact with the top hBN flake at 40 °C, using xyz micromanipulators so that the contact front between stamp and substrate covers the hBN flake. Stamps are then retracted, and the hBN flake in contact with the PC is picked up from the substrate. 1L-WSe_2 on PMMA is then aligned to the top hBN flake on the PC stamp and picked up at 80 °C, before the bottom hBN flake is then picked up at 40 °C. The completed ML-hBN/1L-WSe_2/ML-hBN heterostructure is then transferred onto a 285 nm SiO_2/Si substrate held at 180°C, before the PC residue is removed by immersion in chloroform and then ethanol for 30mins.[5] The hBN thickness is measured with a Bruker Dimension Icon atomic force microscope in tapping mode, finding thicknesses of 15 nm and 10 nm for bottom and top layer, respectively.

Optical measurements

The transient reflectivity microscopy setup is powered by an amplified Ti:sapphire laser generating 100fs pulses at 800 nm (1.55 eV) with 2mJ energy and 2kHz repetition rate. A fraction of the laser output is used to drive a non-collinear optical parametric amplifier (NOPA) pumped at 400 nm (3.1eV) by the second harmonic of the laser, generating broadband visible pulses.[6] The chirp in the pump pulses, mainly due to dispersive glass elements (lenses, beam splitters, filters) present in the setup, is compensated by using chirped mirrors,[7] which compress the NOPA pulses down to$\sim$50fs. The pump pulses are modulated by a mechanical chopper at 500Hz. For the broadband probe pulses, a white-light continuum is generated by focusing the 800nm laser output on a 1-mm-thick sapphire plate.[8] The probe beam is sent to a mechanical delay line which controls the delay between pump and probe pulses. The pump and probe pulses are then collinearly combined by a 1-mm-thick

beam splitter and focused on the sample using an objective lens with 8mm focal length (NA=0.3). Diameter of the probe at the sample position is about 3 μm; the pump diameter is slightly larger. The sample is mounted in a closed-cycle He cryostat and kept at 8K. A CMOS camera is used to acquire a wide field image of the sample and to spatially align pump and probe pulses on a selected region of the sample. The reflected probe beam is collected by the objective lens and delivered to a dispersive spectrometer with a CCD camera (Princeton Instruments PIXIS 100). Pixel binning is used to speed up the acquisition time of the camera. We achieve a sensitivity of approximately of 10^{-4} for the measured differential signal by consequently acquiring and averaging over 300 pairs of spectra at a fixed delay time with and without pump excitation, and by averaging the pump-probe maps over multiple (i.e. 20-30) time scans. The setup is also equipped with a CW 532 nm diode laser and a fiber-coupled broadband tungsten white lamp for the static optical characterization of the sample (PL and RC). For pump-probe and PL measurements, a set of long-pass (LP650) and short-pass (SP600) filters is used to cut out the excitation light from the signal.

Transfer Matrix analysis

In order to extract the spectral and temporal evolution of the exciton and trion peaks from the $\Delta R/R$ maps, we followed the analysis reported in Refs. 9,10. The transient reflectivity $R(\omega, \tau)$ of 1L-WSe_2 at each delay time is determined by combining the equilibrium Reflectivity $R(\omega)$ and the transient reflectivity maps $\Delta R/R$ reported in fig. 2 of the main text:[10]

$$R(\omega, \tau) = R(\omega) \left[\tfrac{\Delta R}{R}(\omega, \tau) + 1 \right]$$

The static reflectivity $R(\omega)$ has been retrieved by fitting the static RC spectrum. In the fitting procedure, the dielectric function of the TMD is modeled by a Kramers-Kronig constrained variational method[9–11] while the interference effects of the multiple reflections from the interfaces of the heterostructure are modeled by the TTM. This procedure allows one to retrieve also the static absorption spectrum which is reported in Fig. 3. By repeating the same fitting procedure on the out-of-equilibrium data we determine the non-equilibrium absorption spectra as a function of the delay time around the ground and excited state excitons (see Fig. 3 c-d). The temporal evolution of the trion and exciton oscillator strengths are then determined from the time-dependent absorption $\alpha(\omega, \tau)$, fitted with Lorentzians. In particular, Fig.4a and Figs.5a,b of the main text report the

temporal dynamics of the variations of the integrated oscillator strength of A and A* for 1s and 2s states, with respect to their values at equilibrium ($\Delta\alpha$).

Theoretical calculations

We model the exciton states in 1L-WSe_2 by means of a Wannier equation:[12]

$$-\frac{\hbar^2}{2\mu}\Delta\psi(r) - V(r)\psi(r) = E\psi(r), \tag{S1}$$

with Keldysh-Rytova[13,14] screened interaction potential:

$$V(r) = \frac{e^2}{4\pi\varepsilon_0}\frac{\pi}{2r_0}\left[H_0\left(\frac{\kappa r}{r_0}\right) - Y_0\left(\frac{\kappa r}{r_0}\right)\right]. \tag{S2}$$

Here ϵ_0 is the vacuum permittivity, $\mu = 0.21m_0$[15] is the exciton reduced mass in units of free electron mass, m_0, H_0 and Y_0 are the Struve and the Bessel functions of second kind, respectively, $\kappa = 4.5$ is the dielectric screening by hBN, $r_0 = 4.1$nm is the 1L-WSe_2 screening length. Setting the bandgap $E_g = 1.897$eV, we recover the experimental $E_{A1s} = E_g - |E_{1s}| = 1.73$eV, and $E_{A2s} = E_g - |E_{2s}| = 1.86$eV of $A1s$ and $A2s$ exciton resonances. The wave functions are used to calculate the exciton radiative lifetimes as:[16]

$$\frac{1}{\tau_i^{\mathrm{r}}} = \frac{1}{4\pi\varepsilon_0}\frac{2\pi g E_{X,i}}{\sqrt{\kappa}\hbar^2 c}|d_{cv}|^2|\psi_i(r=0)|^2, \tag{S3}$$

where $i = \{A1s, A2s\}$, $g = 4$ is the spin-valley degeneracy rate, $d_{cv} = 7$ D is the dipole matrix element of interband transition, treated as a fitting parameter. We get $\tau^{\mathrm{r}}_{A2s}/\tau^{\mathrm{r}}_{A1s} \sim |\psi_{A1s}(r = 0)|^2/|\psi_{A2s}(r=0)|^2 \sim 10$. The non-radiative lifetime of excitons is attributed to quasi-elastic scattering on acoustic phonons, and within the Fermi golden rule can be evaluated as:[17]

$$\frac{1}{\tau_i^{\mathrm{nr}}} = \frac{2\pi}{\hbar}\sum_{\mathbf{k}',\mathbf{q}}|M^{\mathbf{q}}_{\mathbf{k},\mathbf{k}',i}|^2(1+2n_q)\delta(E_k - E'_k), \tag{S4}$$

where $E_k = \hbar^2k^2/(2M)$ is the exciton center-of-mass energy, $n_q \approx k_{\mathrm{B}}T/(\hbar v_s q)$ is the thermal occupation of phonons with wave vector q, M is the exciton total mass, k_{B} is the Boltzmann

constant, $v_s = 3.3 \times 10^3$ m/s is the sound velocity.[18] The scattering matrix element reads

$$M^{\mathbf{q}}_{\mathbf{k},\mathbf{k}',i} = \sqrt{\frac{\hbar}{2\rho_{2\mathrm{D}} q v_s S}} q(D_c - D_v)\mathcal{F}_i(q)\delta_{\mathbf{k},\mathbf{k}'+\mathbf{q}}, \tag{S5}$$

where $\rho_{2\mathrm{D}} = 6.04 \times 10^{-6}$kg/m^2 is the 1L-WSe$_2$ mass density, S is the normalization area, D_c, D_v are the deformation potentials for the conduction and valence bands, respectively, with $|D_c - D_v| \sim$ 3.7eV, and the form factor

$$\mathcal{F}_i(q) = \int e^{-i\mathbf{q}\cdot\mathbf{r}/2} |\psi_i(r)|^2 \mathrm{d}^2 r. \tag{S6}$$

The spatial extent of the exciton wave function can be estimated via the parameter $a_i = \langle \psi_i | r | \psi_i \rangle$, corresponding to Bohr radius for the ground state. The relevant wave vector range within the optically active region can be defined from the exciton light cone edge condition: $E_i + \hbar^2 k^2_{\mathrm{max}}/(2M_X) = \hbar c k_{\mathrm{max}}/\kappa$, so that $k < 0.05$ nm^{-1}. Thus, for the wave vectors of interest $k \sim q \ll 1/a_i$ one has $\mathcal{F}_i(q) \approx 1$, as it follows from Fig. S1. Therefore, the direct evaluation yields in

$$\frac{1}{\tau_i^{\mathrm{nr}}} = \frac{M(D_c - D_v)^2 k_{\mathrm{B}} T}{\rho_{2\mathrm{D}} v_s^2 \hbar^3} \tag{S7}$$

both for $A1s$ and $A2s$ states.

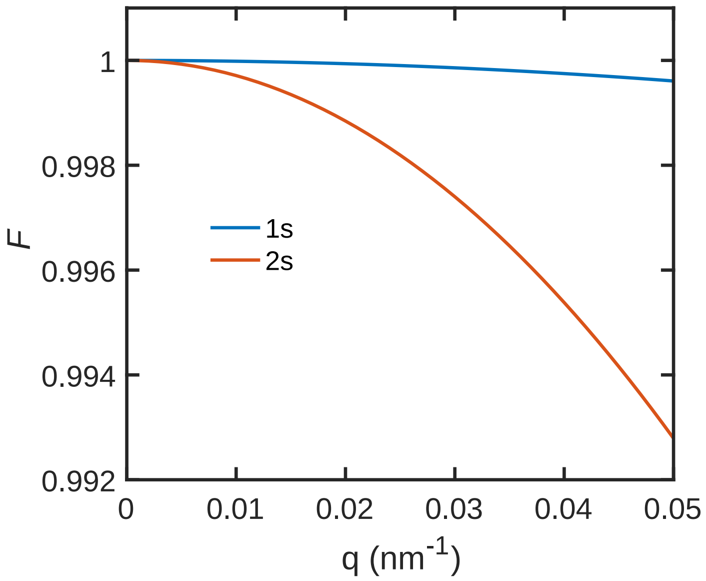

Supplementary Figure S1: Calculated form factor for 1s (blue line) and 2s excitons (red line) as a function of the wavevector

The temporal dynamics of exciton non-equilibrium population n_i under pulsed excitation in the linear regime can be modeled as:[19]

$$\frac{\mathrm{d}n_i}{\mathrm{d}t} = Pe^{-\frac{(t-t_0)^2}{2\delta t^2}} - \frac{n_i}{\tau_i}, \tag{S8}$$

where P characterizes the pump intensity, $\delta t = 50$ fs is the pulse duration, and $t_0 = 10$fs is the time at which the pump pulse intensity peaks. The simulations are in Fig.4c of the main text, where $\Delta R/R \propto n_i(t)$.

Fit of the dynamics

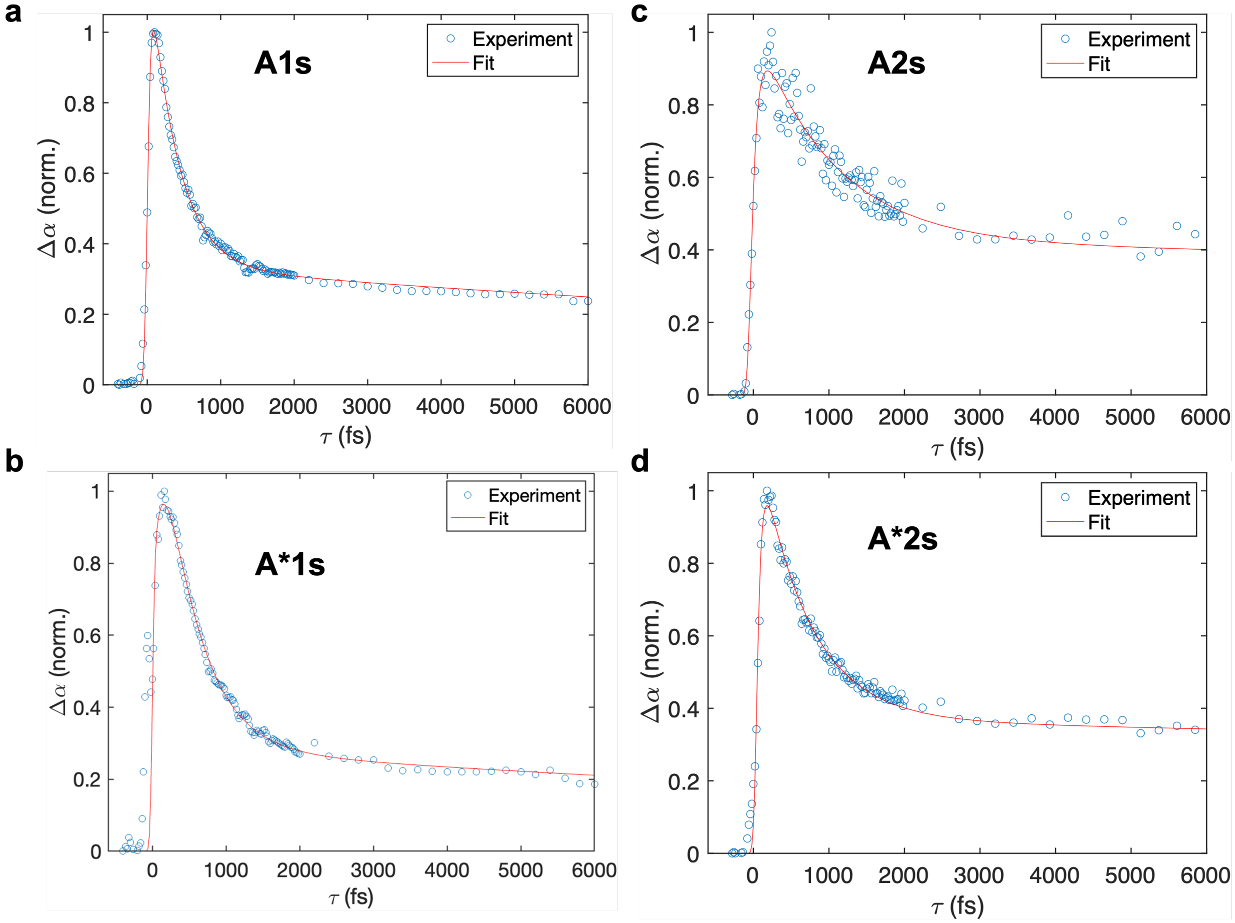

Supplementary Figure S2: Experimental temporal dynamics of A1s **(a)**, A*1s **(b)**, A2s **(c)** and A*2s **(d)** fitted with multi-exponential functions (red lines).

In order to extract the relevant lifetimes from the transient reflectivity maps in Fig.2 of the main text, we fitted the integrated absorption intensity variation ($\Delta\alpha$) of Rydberg excitons and trions, extracted from the TMM analysis, using multi-exponential functions. We include in the fit a rise and two decay components, convoluted with a Gaussian taking into account the Instrument Response Function (IRF) related to the pump pulse duration, which was considered as a fixed fitting parameter. The fitted values for the rise times were all comparable with the experimental uncertainties, therefore we can consider the buildup as instantaneous and we do not report its time constant. The fitted decay times (with the errors from the fit) are displayed in the table below, while Fig. S2 shows the comparison between experimental and fitted curves.

	A1s	A*1s	A2s	A*2s
τ_{fast} [fs]	366 ± 22	475 ± 240	1021 ± 114	652 ± 39
τ_{slow} [ps]	19.7 ± 2.3	19 ± 20	96.3 ± 19.2	75.3 ± 7.7

Additional pump-probe experiments

We probed the dynamic behaviour of A2s states in our WSe_2 sample, pumping directly A1s excitons at lower energies (1.7 eV), and comparing the results with the experiments reported in the main text with above-bandgap pump (2.34 eV) at the same fluence. Fig. S3b shows spectral cross-sections of the transient reflectivity maps for the two cases, taken at 100 fs delay time, where a similar shape is observed with a more pronounced negative part for the low pump photon energy measurement, related to photo-induced trions formation. This probably indicates that the binding process of A2s excitons with charges is subsequent to the formation of A1s, which start immediately to dissociate when directly pumped, creating free charges due to Auger-like effects.

Plotting the temporal dynamics of the transient reflectivity traces for A2s (Fig.S3 c,d), we observe an ultrafast build-up of the signal in the low pump photon energy case, similar to the above-bandgap excitation, and a slower and more prominent second decay. The former evidence demonstrates that A1s and A2s share the same ground state. The slower decay instead suggests that a large amount of upconverted or Auger excitons populate dark states and then back-scatter in the bright Rydberg states, forming an incoherent population which recombines on a longer timescale. Plotting the temporal dynamics of the transient reflectivity traces for A2s (Fig.S3 c,d), we observe an ultrafast build-up of the signal in the low pump photon energy case, similar to the above-bandgap excitation, and a slower and more prominent second decay. The former evidence demonstrates that A1s and A2s share the same ground state. The slower decay instead suggests that a large amount of upconverted or Auger excitons populate dark states and then back-scatter in the bright Rydberg states, forming an incoherent population of excitons which recombine in longer times.

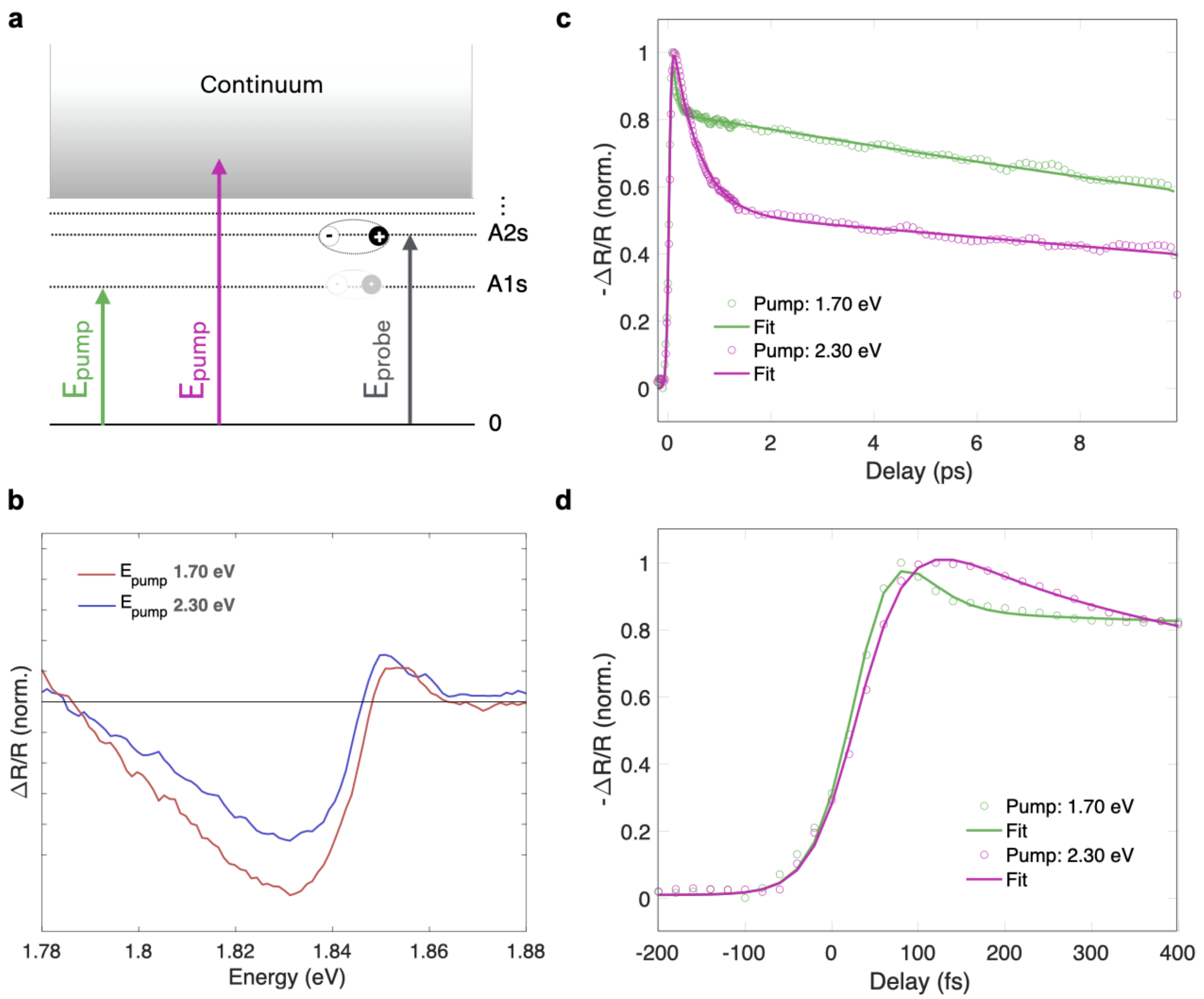

Supplementary Figure S3: **a)** Sketch of the pump and probe pulse energies for the low energy pump experiments. **b)** Spectral cross-sections of A2s transient reflectivity at 100 fs delay time, when pumped at 1.7 eV (red) or 2.4 eV (blue). **c)** Experimental temporal dynamics (circles) of A2s with different pump configurations and related fit (lines). **d)** Zoomed view of **(c)** showing the dynamics of A2s up to 400 fs.

References

(1) Taniguchi, T. & Watanabe, K. Synthesis of high-purity boron nitride single crystals under high pressure by using Ba–BN solvent. *Journal Of Crystal Growth*. **303**, 525-529 (2007)

(2) Novoselov, K., Jiang, D., Schedin, F., Booth, T., Khotkevich, V., Morozov, S. & Geim, A. Two-dimensional atomic crystals. *Proceedings Of The National Academy Of Sciences*. **102**, 10451-10453 (2005)

(3) Zhang, X., Lou, F., Li, C., Zhang, X., Jia, N., Yu, T., He, J., Zhang, B., Xia, H., Wang, S. & Others Flux method growth of bulk MoS_2 single crystals and their application as a saturable absorber. *CrystEngComm.* **17**, 4026-4032 (2015)

(4) Casiraghi, C., Hartschuh, A., Lidorikis, E., Qian, H., Harutyunyan, H., Gokus, T., Novoselov, K. & Ferrari, A. Rayleigh imaging of graphene and graphene layers. *Nano Letters.* **7**, 2711-2717 (2007)

(5) Purdie, D., Pugno, N., Taniguchi, T., Watanabe, K., Ferrari, A. & Lombardo, A. Cleaning interfaces in layered materials heterostructures. *Nature Communications.* **9**, 5387 (2018)

(6) Cerullo, G., Nisoli, M., Stagira, S. & De Silvestri, S. Sub-8-fs pulses from an ultrabroadband optical parametric amplifier in the visible. *Optics Letters.* **23**, 1283-1285 (1998)

(7) Manzoni, C., Polli, D. & Cerullo, G. Two-color pump-probe system broadly tunable over the visible and the near infrared with sub-30 fs temporal resolution. *Review Of Scientific Instruments.* **77**, 023103 (2006)

(8) Alfano, R. The supercontinuum laser source: the ultimate white light. (Springer,2016)

(9) Raja, A., Waldecker, L., Zipfel, J., Cho, Y., Brem, S., Ziegler, J., Kulig, M., Taniguchi, T., Watanabe, K., Malic, E. & Others Dielectric disorder in two-dimensional materials. *Nature Nanotechnology.* **14**, 832-837 (2019)

(10) Trovatello, C., Katsch, F., Li, Q., Zhu, X., Knorr, A., Cerullo, G. & Dal Conte, S. Disentangling many-body effects in the coherent optical response of 2D semiconductors. *Nano Letters.* **22**, 5322-5329 (2022)

(11) Kuzmenko, A. Kramers–Kronig constrained variational analysis of optical spectra. *Review Of Scientific Instruments.* **76** (2005)

(12) Haug, H. & Koch, S. Quantum theory of the optical and electronic properties of semiconductors. (world scientific,2009)

(13) Rytova, N. Screened potential of a point charge in a thin film. *Proc. MSU, Phys. Astron..* **22**, 30-37 (1967)

(14) Keldysh, L. Coulomb interaction in thin semiconductor and semimetal films. *J. Exp. Theor. Phys.*. **29** pp. 658 (1979)

(15) Goryca, M., Li, J., Stier, A., Taniguchi, T., Watanabe, K., Courtade, E., Shree, S., Robert, C., Urbaszek, B., Marie, X. & Others Revealing exciton masses and dielectric properties of monolayer semiconductors with high magnetic fields. *Nature Communications*. **10**, 4172 (2019)

(16) Andreani, L., Tassone, F. & Bassani, F. Radiative lifetime of free excitons in quantum wells. *Solid State Commun.*. **77**, 641-645 (1991)

(17) Shree, S., Semina, M., Robert, C., Han, B., Amand, T., Balocchi, A., Manca, M., Courtade, E., Marie, X., Taniguchi, T. & Others Observation of exciton-phonon coupling in $MoSe_2$ monolayers. *Physical Review B*. **98**, 035302 (2018)

(18) Jin, Z., Li, X., Mullen, J. & Kim, K. Intrinsic transport properties of electrons and holes in monolayer transition-metal dichalcogenides. *Phys. Rev. B*. **90**, 045422 (2014)

(19) Kavokin, A., Baumberg, J., Malpuech, G. & Laussy, F. Microcavities. (Oxford university press, 2017)
